# MRTF-A-mediated protection against amyloid-β-induced neuronal injury correlates with restoring autophagy via miR-1273g-3p/mTOR axis in Alzheimer models

**DOI:** 10.18632/aging.203883

**Published:** 2022-05-23

**Authors:** Wei Zhang, Yuewang Yang, Zifei Xiang, Jinping Cheng, Zhijun Yu, Wen Wang, Ling Hu, Fuyun Ma, Youping Deng, Zhigang Jin, Xiamin Hu

**Affiliations:** 1 Affiliated Wuhan Resources and Wisco General Hospital, University of Science and Technology, Wuhan, Hubei, China; 2 College of Pharmacy, Shanghai University of Medicine and Health Sciences, Shanghai, China; 3 College of Medicine, Wuhan University of Science and Technology, Wuhan, Hubei, China; 4 Bioinformatics Core Department of Quantitative Health Sciences, John A. Burns School of Medicine, University of Hawaii, Honolulu, HI 96813, USA

**Keywords:** Alzheimer’s disease, autophagy, miR-1273g-3p, MRTF-A, mTOR

## Abstract

Myocardia-Related Transcription Factors-A (MRTF-A), which is enriched in the hippocampus and cerebral cortex, has been shown to have a protective function against ischemia hypoxia-induced neuronal apoptosis. However, the function of MRTF-A on β-amyloid peptide (Aβ)-induced neurotoxicity and autophagy dysfunction in Alzheimer’s disease is still unclear. This study shows that the expression of MRTF-A in the hippocampus of Tg2576 transgenic mice is reduced, and the overexpression of MRTF-A mediated by lentiviral vectors carrying MRTF-A significantly reduces the accumulation of hippocampal β-amyloid peptide and reduces cognition defect. Overexpression of MRTF-A inhibits neuronal apoptosis, increases the protein levels of microtubule-associated protein 1 light chain 3-II (MAP1LC3/LC3-II) and Beclin1, reduces the accumulation of SQSTM1/p62 protein, and promotes autophagosomes-Lysosomal fusion *in vivo* and *in vitro*. Microarray analysis and bioinformatics analysis show that MRTF-A reverses Aβ-induced autophagy impairment by up-regulating miR-1273g-3p level leading to negative regulation of the mammalian target of rapamycin (mTOR), which is confirmed in Aβ_1-42_-treated SH-SY5Y cells. Further, overexpression of MRTF-A reduces Aβ_1-42_-induced neuronal apoptosis. And the effect was abolished by miR-1273g-3p inhibitor or MHY1485 (mTOR agonist), indicating that the protection of MRTF-A on neuronal damage is through targeting miR-1273g-3p/mTOR axis. Targeting this signaling may be a promising approach to protect against Aβ-induced neuronal injury.

## INTRODUCTION

Alzheimer’s disease is a common progressive neurodegenerative disorder characterized by cognitive dysfunction. The pathological features of Alzheimer’s disease include senile plaques composed of amyloid-beta and neurofibrillary tangles, accompanied by the loss of neurons and synapses in the brain and the proliferation of glial cells [1]. Although its etiology is unknown, assumptions of pathogenesis are proposed, such as β-amyloid peptide accumulation, neurons apoptosis, genetic mutations, synaptic dysfunction, etc. [2–5]. The brains of AD patients display considerable, pathological β-amyloid peptide and presenilin1 protein [6, 7].

The myocardin and myocardin-related transcription factors (MRTF-A and -B), as a co-activator of serum response factor (SRF), synergistically activates the transcription of a subset of genes involved in cytoskeletal organization and muscle cell differentiation [8]. MRTF-A gene expression is enriched in forebrain, especially in hippocampus and cerebral cortex [9, 10], which plays an important role of normal brain development. MRTF-A mutant mice exhibited abnormal neuronal migration and impaired neurite outgrowth, as well as decreased expression and activity of actin and actin-severing proteins gelsolin and cofilin [11]. Our previous study has shown that MRTF-A attenuates hydrogen peroxide-induced and Aβ-induced neuronal injury in primary cortical neuron by upregulating Bcl-2 and Mcl-1 through triggering their CArG box [12, 13]. However, the role of MRTF-A in the regulation of AD progression has not been systematically illustrated. Based on the miRNA microarray analysis, we found that miR-1273g-3p might involve in the protection of MRTF-A on Aβ-induced neuronal injury. However, whether MRTF-A affect the AD progression via regulating miR-1273g-3p is unknown.

MicroRNA represents an evolutionarily conserved 20-23 nucleotide group [14]. Studies have shown that some miRNAs seem to be involved in the AD process, which create highly complex and interactive regulatory miRNA-mRNA networks in an independent, coordinated and/or cooperative manner inducing to changes in atypical mRNA abundance, gene expression, pro-inflammatory signals, synapses, and amyloidosis [14–16]. The main mode of miRNA action is to recognize the 3’ untranslated region (3’-UTR) of a specific messenger RNA (mRNA) target through base pair complementation [14]. Using the bioinformatic analysis, we found a Homo sapiens (Hsa) miR-1273g-3p binding site exists in the 3’-UTR of the mammalian target of rapamycin (mTOR), predicting that miR-1273g-3p might negatively regulate mTOR expression.

mTOR is a conserved serine/threonine protein kinase, which belongs to the phosphoinositide-3-kinase (PI3K)-related kinase family of protein kinases [17]. Some small molecules, including small non-coding RNA (sncRNA) molecules, can regulate autophagy through mTOR-dependent or independent pathways [18, 19], and are shown a protective effect in neurodegenerative disease models [20, 21], suggesting the mTOR pathway is one of the most promising targets for AD treatment via regulating autophagy [22]. Therefore, we speculated that mTOR may be involved in the effect of MRTF-A on Aβ-induced neuronal injury via miRNA-mRNA interaction with miR-1273g-3p.

This study was to investigate the effect of MRTF-A on Aβ-induced neurotoxicity in the models of Tg2576 AD mouse and Aβ_1-42_-treated SH-SY5Y cell. According to the hypothesis of MRTF-A regulating miR-1273g-3p/mTOR axis, we further investigated the mechanism of MRTF-A based on bioinformatics analysis and experimental verification. Our results might provide new avenues for therapeutic approaches of AD.

## MATERIALS AND METHODS

### Construction of lentivirus carrying MRTF-A and microinjection

To investigate the effect of MRTF-A on Tg2576 mice, the GV287 lentivirus (GOSL29806, Genechem Co., Ltd.) mediating MRTF-A overexpression (LV-MRTF-A) was constructed. Mouse MRTF-A cDNA (NM001082536) was amplified by PCR: MRTF-A Forwards: GAGGATCCCCGGGTACCGGTCGCCACCATGCCGCCTTTGAAAAGCCCCGCTG; MRTF-A Reverse: TCCTTGTAGTCCATACCCAAGCAGGAATCCCAGTGGAGCTGC). Subsequently, insert the PCR product into the parent vector Ubi-MCS-3FLAG-SV40-EGFP (Genechem Co., Ltd, China) between the Age I and BamH I sites, and empty as negative control (LV-NEG) [23]. The lentivirus was produced by co-transfected with a transfer vector and three packaging vectors (pGC-LV vector 20 μg, pHelper-1.0 vector 15 μg, pHelper-2.0 vector 10 μg), and then purified by ultracentrifugation, and its titer was determined via fluorescence.

Using a microinjection system, mice is anesthetized and placed in a stereotactic device (Zhenghua Biological instrument Equipment Co. Ltd.), afterward the mice were bilateral injected with 4.0 μL of purified virus into the lateral ventricle (-2.5 mm dorsal/ventral, -1.0 mm lateral, and -0.22 mm anterior/posterior to bregma) [24]. The transfection efficiency *in vivo* was measured by western blot analysis after one month of injection.

### Animals and treatment

Tg2576 mice carrying human APP695 with Swedish double mutations (hAPP; HuAPP695; K670N/M671L) were gifted by Dr. Zeng Yan (Wuhan University of Science and Technology, China). Wild-type male C57BL mice (Certificate No: SCXK (Q)2015-0018) were purchased from the Hubei Provincial Center for Disease Control and Prevention. All mice were allowed free access to food and water before the procedure was performed under optimal conditions (12/12 hours light/dark with humidity at 60 ± 5% and temperature at 22 ± 3° C) [25].

**Figure 1:**
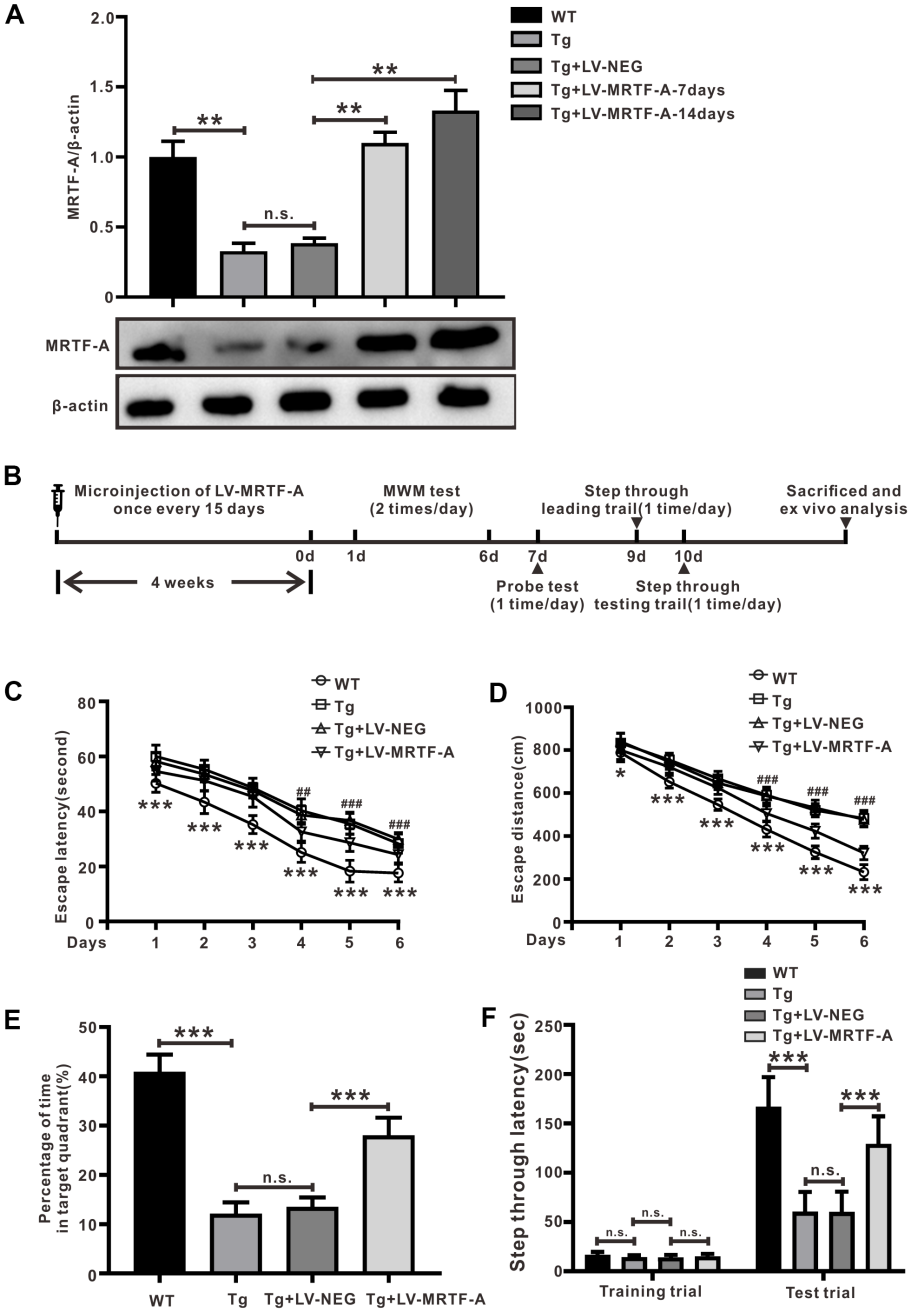
**MRTF-A alleviated cognitive impairment in Tg2576 mice.** (**A**) MRTF-A expression in the hippocampus of WT and Tg2576 mice was analyzed after transfected with or without LV-MRTF-A. Data represent means ± SEM from 5 mice per group. (**B**) The diagram of the treatment of LV-MRTF-A and the assessment of cognitive function in Tg2576 mice. (**C**–**F**) The water maze test (**C**, **D**), the probe test (**E**) and the step-through type passive avoidance test (**F**) were carried out in WT or Tg2576 mice. Data represent means ± SEM from 14-12 mice per group, *P < 0.05, **P < 0.01 and ***P < 0.001 (two-way ANOVA, Tukey’s Multiple Comparison Test).

Mice (about 6.5 months old) were randomly divided into 3 groups: (i) Tg2576 mice microinjected with LV-MRTF-A (n=14); (ii) Tg2576 mice microinjected with LV-NEG (n=14); (iii) Age-matched C57BL/6 (WT) mice microinjected with normal saline (n=12). Microinjection of LV-MRTF-A, LV-NEG or normal saline were treated once every 2 weeks for 6 weeks, including 2 weeks of behavioral test [26]. The experimental arrangement is summarized in Figure 1B. After behavioral evaluations, the mice were sacrificed and the brains were taken off for the analysis of western blot, immunohistochemical, Nissl staining and transmission electron microscope.

### Behavioral test

The behavioral test was performed as described previously. Morris water maze (MWM) test was carried out in a circular plastic pool filled with opaque water of skimmed milk kept at 25 ± 1° C. The escape platform was submerged 1-1.5cm below the water surface. The time spent by each mouse to locate the platform (escape latency) and its track were recorded by a camera (Panlab, Barcelona, Spain). After 24 hours of MWM test, a memory test (probe test) was performed by removing the platform. The mice were allowed to swim freely for 60 s. The retention ability of memory was estimated by the time spent in the target quadrant area [27].

Passive avoidance performance test was performed in two compartments (light and dark compartments) (Med Associates Inc., Vermont, USA) [26]. At the beginning, the mice were placed in an illuminated compartment for training. And then the mice were moved into the dark compartment and received an electric shock. Then the mice were returned to their cage. One day later, the mice were placed in the illuminated compartment, and the latency to enter the dark compartment was measured, described as step-through latency.

### Nissl staining

After neurobehavioral evaluation, mice (3 animals/group) were anesthetized, and then the brain was removed. The paraffin-embedded mouse hippocampus was cut into 4 mm thick sections. After deparaffinization and rehydration, the sections were stained in Nissl staining solution at 60° C for 30 minutes (C0117, Beyotime Biotechnology, Shanghai, China). The sections are dehydrated with absolute ethanol, xylene is transparent and sealed with neutral gum. The morphology was observed using a multifunctional digital pathology system (APERIO VERSA 8, Leica, Germany).

### Transmission electron microscope analysis

Autophagic vacuoles were assessed by electron microscopy. After heart perfusion with glutaraldehyde, hippocampal CA1 area was separated and fixed in 4 % paraformaldehyde for 2 hours then embedded with epoxy resin. The samples were sliced into 90 nm ultrathin sections and observed under HZ600 transmission electron microscope (Hitachi, Japan).

### Immunofluorescence staining

The immunofluorescence staining of brain paraffin sections were performed as previously described [25]. After blocking with 5% bovine serum albumin, the sections were incubated with anti-MRTF-A (1:100, sc-398675, Santa Cruz, CA, USA), rabbit anti-β-amyloid (Cell Signaling Technology, 1:2000) or rabbit anti-LC3B (1: 500, ab48394, Abcam, MA, USA), respectively. Then, the sections were washed with 0.1M PBS and then incubated with the fluorescence-labeled secondary antibodies (goat anti-mouse/rabbit 1:200, ab150113, ab150077, Abcam, MA, USA) for 30 minutes at 37° C, and then washed with PBS. Next, the nuclei were stained with DAPI (5 μg/ml) for 2 minutes, and then analyzed with a laser scanning confocal microscope (CX31-32RFL, Olympus).

### Cell culture and treatment

Human SH-SY5Y cells were grown in in DMEM high glucose with 10% fetal bovine serum (G4510, Servicebio, Shanghai, China) and 1% penicillin-streptomycin solution at 37° C in a humidified incubator containing 5% CO_2_. For the effect of MRTF-A on Aβ_1–42_-induced SH-SY5Y cell damage, the cells were firstly transduced with or without LV-MRTF-A (Genechem, Shanghai, China), miR-1273g-3p mimics (Sequence: 5’-ACCACUGCACUCCAGCCUGAG-3’), miR-1273g-3p inhibitors (Sequence: 5’-CUCAGGCUGGAGUGCAGUGGU-3’) and their negative (Ribobio, Guangzhou, China) as control. After 48 hours, the cells were exposed to Aβ_1–42_ (#89298, 10 μM, GL Biochem, Shanghai, China) for 24 hours. In addition, to prove the effect of mTOR involved in MRTF-A against Aβ-induced neuronal damage, MHY1485(5 mM) (an agonist of mTOR; S7811, Selleck, Shanghai, China) and 3-Methyladenine (5 mM) (3-MA; S2767, Selleck, Shanghai, China) were also used to co-incubate in the cells for 24 hours, and then exposed to 10 μM Aβ_1–42_.

### Cell viability assay

SH-SY5Y cells were seeded into 96-well plates at a density of 8×10^3^ cells per well. Transfection of LV-MRTF-A or its negative control for 48 hours, and then incubated with or without 10 μM of Aβ_1-42_ at 37° C for 24 hours. 20 μL of 3-(4,5-dimethylthiazole-2-yl)-2,5-diphenyl tetrazolium bromide (5 mg/mL, M2128, Sigma-Aldrich St. Louis, USA) was added to each well for 4 hours at 37° C in darkness. After removing the supernatant, 150 μL of dimethyl sulfoxide was added to dissolve the crystalline formazan. The absorbance was measured using a microplate reader at 490 nm (Synergy 4, Omega Bio-tek, Inc., Norcross, GA, USA).

### TUNEL labeling

The cultured cells were labeled with the TUNEL Bright Red Apoptosis Detection Kit System (A113-01/02/03, Vazyme Biotech, China). Specimens were viewed on a CX31-32RFL confocal microscope. The cells undergoing apoptosis and the total number of cells were measured (Image J software). The apoptotic ratio was apoptotic cells/total cells 100% [28].

### Real-time RT-PCR analysis

miR-1273g-3p expression was determined using the Bulge-Loop miRNA qRT-PCR Primer Set (MQPS0002237-1-100, RiboBio, China) [29]. Amplification was carried out starting with an initial step of 3 min at 95° C, followed by 40 cycles of the amplification step for miR-1273g-3p and U6(MQPS0000002-1-100). The primer sequences were synthesized by Ribobiotechnology Company (Guangzhou, China).

### Western blotting analyses

The protein expressions of the brain tissues and cell specimens were analyzed as described previously [25]. The primary antibodies included MRTF-A (1:1000, sc-398675, Santa Cruz, CA, USA), APP (#2452, Cell Signaling Technology, MA, USA), and BACE1 (1:1000, sc-33711, Santa Cruz, CA, USA), p62(1:1000, P0067, Sigma-Aldrich St. Louis, USA), Beclin1(1:1000, #3738, Cell Signaling Technology, MA, USA), mTOR (1:1000, ab32028, Abcam, MA, USA), p-mTOR(1:1000, sc-293133, Santa Cruz, CA, USA), and β-actin (1:1000, sc-8432, Santa Cruz, CA, USA). The antibodies were diluted in TBST buffer (50mM TrisHCl, 150mM NaCl, 0.1% Tween-20, pH 7.4) (P0015F, Beyotime, Shanghai, China) and incubated with the PVDF membrane at 4° C overnight. Corresponding horseradish peroxidase (HRP)-conjugated secondary antibodies (1:5000, sc-2004, sc-2005, Santa Cruz, CA, USA) was subsequently incubated with the PVDF membrane for 90 minutes at room temperature. Signal detection was performed with an enhanced chemiluminescent (ECL) reagent (G2020-25ML, Servicbio, Shanghai, China). The luminescent signals were detected by a BioRad ChemiDoc MP system.

### Autophagic flux measurement

Cells were cultured in 24-well plates (1×10^5^ cells/well), and then were transfected with the adenovirus of mCherry-GFP-LC3 (20 MOI, multiplicity of infection) at 37° C in 5%CO_2_/95% air. After 24 hours for infection, removed the virus-containing culture medium and then transduced with or without LV-MRTF-A, miR-1273g-3p mimics, miR-1273g-3p inhibitors or their negative, and then exposed to Aβ_1-42_ for 48 hours. The expression of mCherry and GFP was visualized with confocal laser scanning microscopy (Olympus) [30]. The dots of GFP and mCherry were measured in 3 randomly selected areas under fluorescence microscope (Olympus Corporation).

### miRNA microarray

Cells were transduced with or without LV-MRTF-A for 48 hours and then incubated with 10 μM Aβ_1-42_. 24 hours later, total RNA was extracted from cells of each group using Trizol reagent (#15596-026, Invitrogen, Carlsbad, CA, USA). RNA quality was measured using Agilent 2100 Bioanalyzer (Agilent Technologies). Only an RNA integrity number ≥5 was used for further analyses. The coefficient variance (CV) of repeated probe was calculated. The next microarray analysis uses the invariant set normalization method. The miRNAs with expression differences of more than 2.0 times were considered to be significant gene.

### Dual luciferase reporter assay

mTOR cDNA (position 500-800bp) containing mir-1273g-3p predictive binding site of mTOR 3’-UTR (position 684-690) was cloned into pmirGLO dual luciferase miRNA target expression vector (Promega Corporation, Fitchburg, WI, USA) to construct the report vector pmirGLO-mTOR-wt (wild-type). The mutant mTOR, containing a point mutation at the binding site of mir-1273G-3p seed region, was inserted into the vector pmirGLO-mTOR-mut (mutant). Transfection of 293T line with miR-1273g-3p mimics or scramble control, or co-transfected with 10 μg of pmir-GLO-wt-mTOR or pmir-GLO-mTOR-mut using Lipofectamine 3000 transfection reagent (L3000015, Thermo Fisher Scientific). 48 hours later, cells were collected and lysed. Then the luciferase activities were determined using Dual-Luciferase® Reporter Assay System (E1910, Promega Corporation).

### Statistical analysis

Data are presented as means ± SEM. Statistical analyses were carried out with SPSS 16.0. A *P* value less than 0.05 was considered to be statistically significant. Differences among ≥3 groups were statistically analyzed by One-way analysis of variance (ANOVA) followed by Tukey’s post hoc test.

## RESULTS

### Upregulating MRTF-A ameliorated cognitive deficits in Tg2576 mice

Tg2576 mice develop cognitive impairment at about 6-12 months of age [31, 32], was used to explore the role of MRTF-A on cognitive impairments. Data showed that MRTF-A endogenous expression in hippocampus of Tg2576 mouse was decreased significantly compared to wild-type (WT) mice (Figure 1A). To verify overexpression of MRTF-A improved cognitive deficit in Tg2576 mice, mice were preinjected with either MRTF-A lentivirus (LV-MRTF-A) or negative control lentivirus (LV-NEG) into their lateral ventricles once every 15 days. Markedly increasing of MRTF-A expression was observed in Tg2576 mice treated with LV-MRTF-A compared with LV-NEG group at 7 and 14 days after injection (Figure 1A). The behavior tests were performed to assess the spatial ability of learning and memory according to the experiment schedule (Figure 1B). Compared with Tg2576 mice alone or Tg2576 mice treated with negative control lentivirus (LV-NEG), overexpression of MRTF-A reduced the average escape latency and swimming distance of Tg2576 mice, especially at the day 5 or 6 (Figure 1C, 1D). Transfection of LV-MRTF-A prolonged the time in the target quadrant significantly in LV-MRTF-A -treated mice compared to that in Tg2576 mice or in Tg2576+LV-NEG mice. Passive avoidance test is performed on the second day after the probe test. There was no significant difference between Tg2576 mice and Tg2576+LV-NEG mice. The average step latency of Tg2576+LV-NEG mice in in the illuminated compartment was 59.70±15.31 seconds, while the average step latency of LV-MRTF-A treated mice was 128.7±28.50 seconds, which was significantly higher than that of the control group (Figure 1F). These results indicate that MRTF-A may be involved in the pathogenesis and development of AD in mice.

### Overexpression of MRTF-A reduced Aβ-induced neurotoxicity in Tg2576 mice

**Figure 2:**
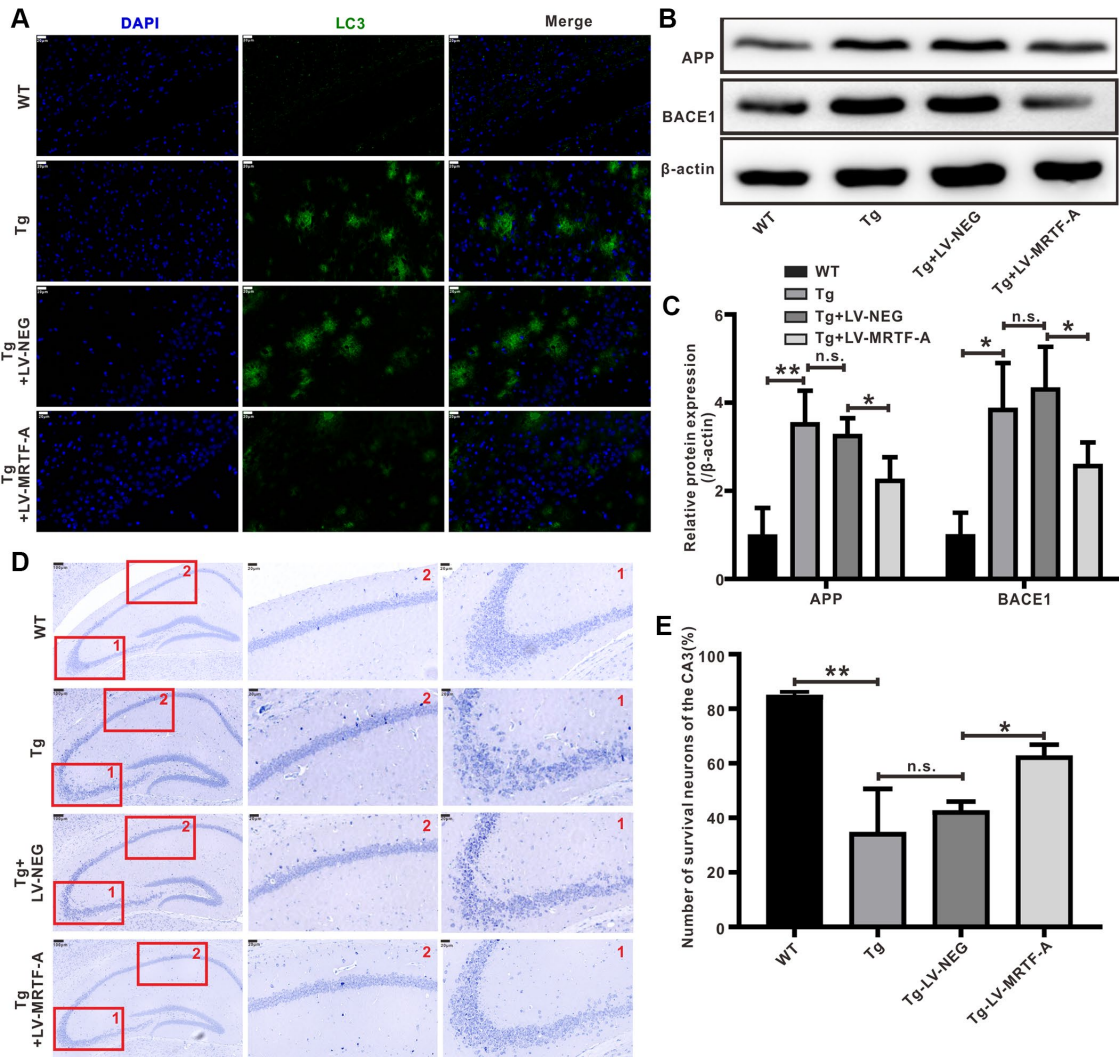
**MRTF-A reduced Aβ-induced neurotoxicity in Tg2576 mouse hippocampus.** (**A**) Representative image of staining with anti-Aβ (green) and DAPI (blue) in the CA1 areas of Tg2576 mice treated with LV-MRTF-A or not. (**B**, **C**) Western blot and quantitative analysis for APP and BACE1 genes. Data are presented as mean ± SEM from 5 mice per group, *P < 0.05 (one-way ANOVA, Tukey’s Multiple Comparison Test). (**D**, **E**) Nissl staining for the neuron morphological changes. The number of survival neurons of CA3 areas was analysis. Data represent means ± SEM from 5 mice per group, *P < 0.05, **P < 0.01 and ***P < 0.001 (one-way ANOVA, Tukey’s Multiple Comparison Test).

The level of insoluble Aβ in the brain of Tg2576 mice increased significantly at about 6 months of age, accumulating into Aβ plaques at 7-8 months of age [33]. Upregulating MRTF-A significantly decreased the Aβ plaque burden (Figure 2A) and the expression of APP and BACE1 compared with Tg2576+LV-NEG group (Figure 2B, 2C). Next, we explored whether MRTF-A reduces Aβ-induced neurotoxicity. Nissl staining showed that compared with WT mice, Nissl body loss, neuron atrophy and nuclear contraction occurred in the hippocampal CA area of Tg2576+LV-NEG mice, accompanied by neuron reduction (Figure 2D, 2E). However, by overexpression of MRTF-A, this morphological change and neuron loss can be reduced (Figure 2D, 2E).

### Overexpression of MRTF-A rescued Aβ-induced autophagy deficit in Tg2576 mice

**Figure 3:**
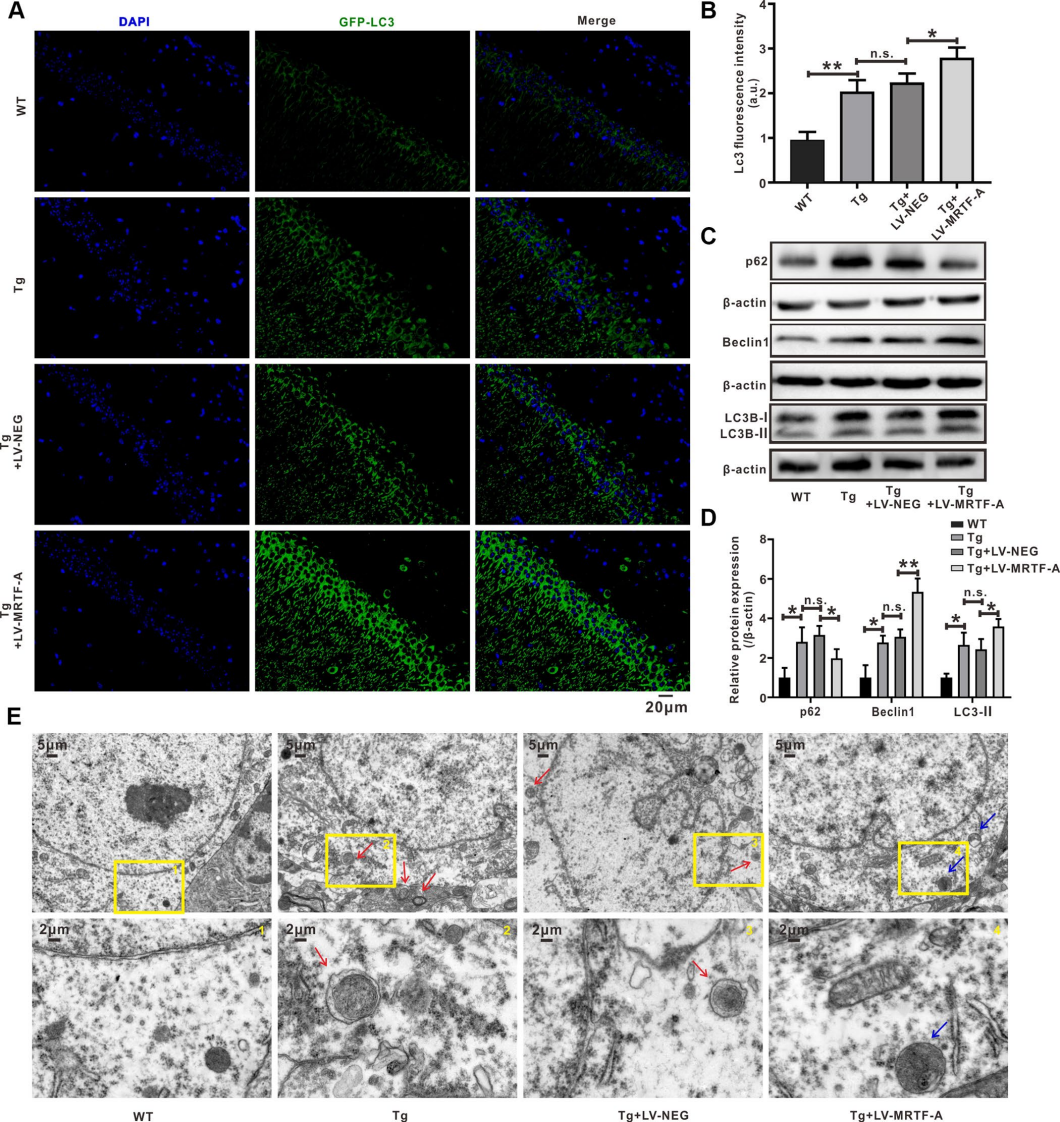
**MRTF-A rescued autophagy deficit in Tg2576 mice.** (**A**–**D**) Immunofluorescence staining and quantitative analysis for LC3B (**A**, **B**); simultaneously western blot and quantitative analysis for Beclin1 and SQSTM1/p62 protein expressions (**C**, **D**). Data represent means ± SEM from 3 mice per group, *P < 0.05, **P < 0.01 and ***P < 0.001 (one-way ANOVA, Tukey’s Multiple Comparison Test). (**E**) Representative transmission electron microscopic images in the hippocampus. Red arrows depict autophagolysosoms and blue arrows depict autolysosomes.

Autophagy plays a crucial role in maintaining intracellular homeostasis, which is considered apro-survival mechanism against stimuli that trigger apoptosis [34, 35]. A defect in autophagosome maturation at the stage of autophagosome-lysosome fusion is an important feature of AD pathology and cognitive dysfunction [36, 37], suggesting a protective role of autophagy against neurodegeneration [38]. Through the construction of co-expression network and node degree analysis, we found that there were some functional and highly connected hubs in MRTF-A, such as ATG5, Beclin1 and so on (Supplementary Figure 1), predicting MRTF-A as an autophagy regulator. MAP1LC3/LC3 is a key regulator of autophagy, which participates in several steps [39]. LC3-II interacts with p62 bodies and thereby facilitate the elongation and closure of autophagosomal membrane [40]. Here, we found that compared with Tg2576 or Tg2576+LV-NEG group, overexpression of MRTF-A significantly increased LC3B fluorescence intensity (Figure 3A, 3B) and Beclin1 protein level, and reduced the expression of p62 protein in hippocampal region (Figure 3C, 3D). Transmission electron microscopy images showed that autophagosomes were rarely noticed in WT mice, while autophagic vacuoles accumulated abnormally in Tg2576 mice and did not fuse with lysosomes. However, overexpression of MRTF-A resulted in an increase in autophagosomes bound to lysosomes, thereby forming autophagolysosomes (Figure 3E), indicating the reversal effect of overexpression of MRTF-A on autophagy defects.

### MRTF-A attenuated Aβ-induced neurotoxicity through regulating autophagy *in vitro*

**Figure 4:**
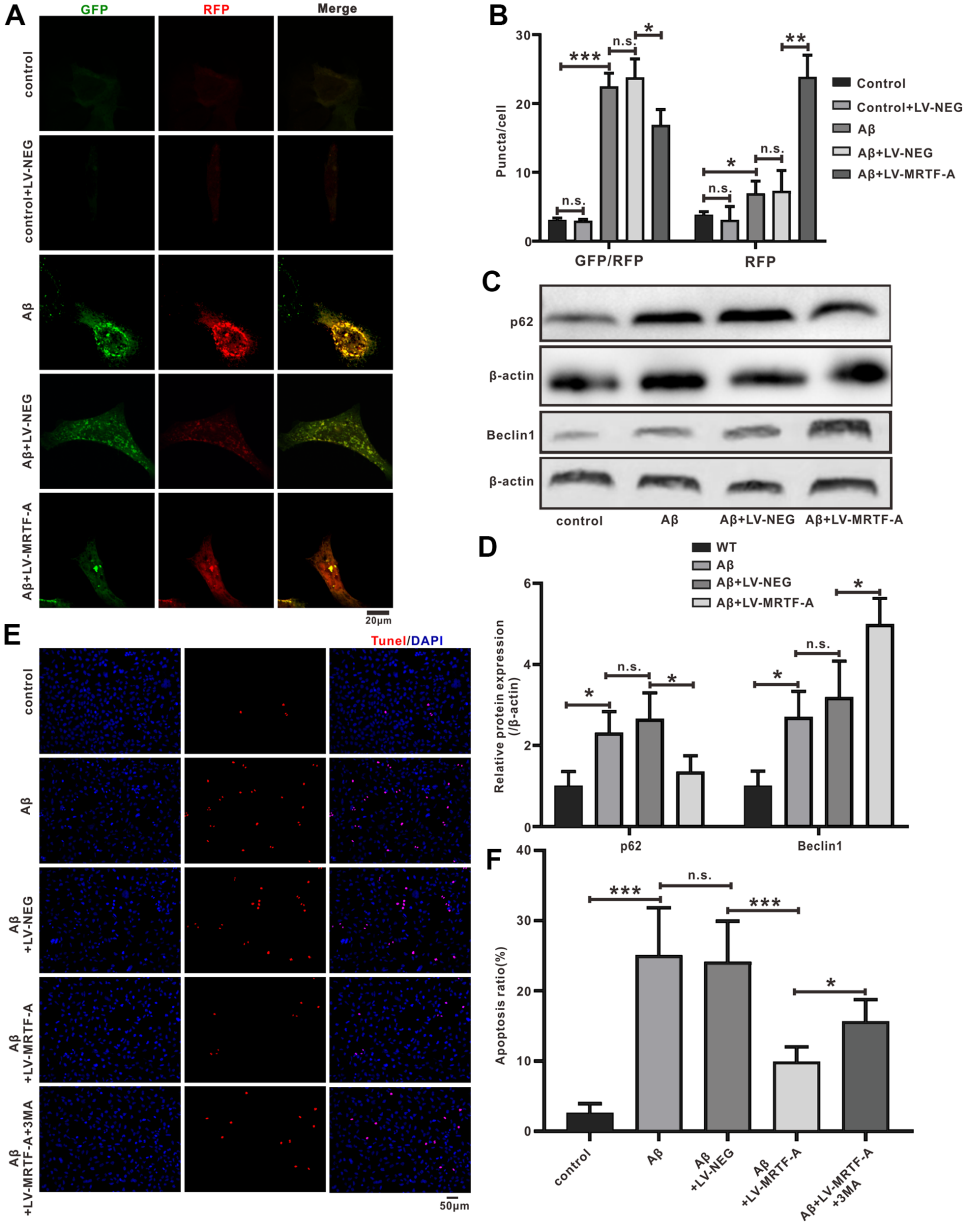
**MRTF-A attenuated Aβ-induced neurotoxicity by promoting autophagy in SH-SY5Y cells.** (**A**, **B**) The autophagy flux analysis using Ad-mCherry-GFP-LC3B-tagged protein. (**C**, **D**) Western blot analysis protein for Beclin1 and p62 expression. (**E**, **F**) TUNEL staining for cell apoptosis in Aβ-treated SH-SY5Y cells followed MRTF-A treatment co-incubation with or without 3-MA. Data represent means ± SEM of 3 independent experiments. *P < 0.05, **P < 0.01 and ***P < 0.001 (one-way ANOVA, Tukey’s Multiple Comparison Test).

The SH-SY5Y cell model treated with Aβ is used to further study the potential neuroprotective effects and mechanisms of MRTF-A. Cells were transfected with LV-MRTF-A or LV-NEG for 48 hours, and then treated with Aβ_1-42_ for 24 hours. Compared with LV-NEG transfected cells, MRTF-A protein expression was confirmed to be significantly increased 48 hours after transfection with LV-MRTF-A (Supplementary Figure 2). To identify whether MRTF-A is responsible for rescuing the impairment of Aβ-induced autophagy flux, the changes of APs and ALs were analyzed by confocal microscopy. As known, the yellow spots in the image (GFP and RFP fluorescence co-location) represent APs, while the red spots alone represent Als. As shown in Figure 4A, 4B, compared with the control group, the yellow and green dots (indicating AP) in the Aβ or Aβ+LV-NEG group were increased significantly, while the red dots (indicating AL) did not increase correspondingly. However, transfection of LV-MRTF-A increased the intensity of red dots in the Aβ+LV-MRTF-A group (Figure 4A, 4B). Consistent with the above results, compared with the Aβ+LV-NEG group, transfection of LV-MRTF-A significantly increased Beclin1 level and decreased SQSTM1/p62 level in Aβ-treated SH-SY5Y cells (Figure 4C, 4D). These results indicate that overexpression of MRTF-A can rescue the impairment of autophagy induced by Aβ_1-42_ in SH-SY5Y cells.

In order to further confirm whether the regulation of autophagy by MRTF-A is responsible for its inhibition of neuronal apoptosis, the cells were incubated with 3-MA (Class III PI3K inhibitor) as an autophagy inhibitor. Then cell apoptosis rates were detected by TUNEL labeling. Compared with the control group, the number of apoptotic neurons in the Aβ or Aβ+LV-NEG group was significantly increased. However, compared with the Aβ+LV-NEG group, the amount of apoptosis in the Aβ+LV-MRTF-A group was significantly reduced. Further, its inhibitory effect was significantly eliminated by co-incubation with 3-MA (Figure 4E, 4F). These results indicate that the inhibition of Aβ-induced neuronal damage by MRTF-A is related to the rescue of autophagy damage.

### Detection and verification of miRNA microarray in Aβ-treated SH-SY5Y cells

**Figure 5:**
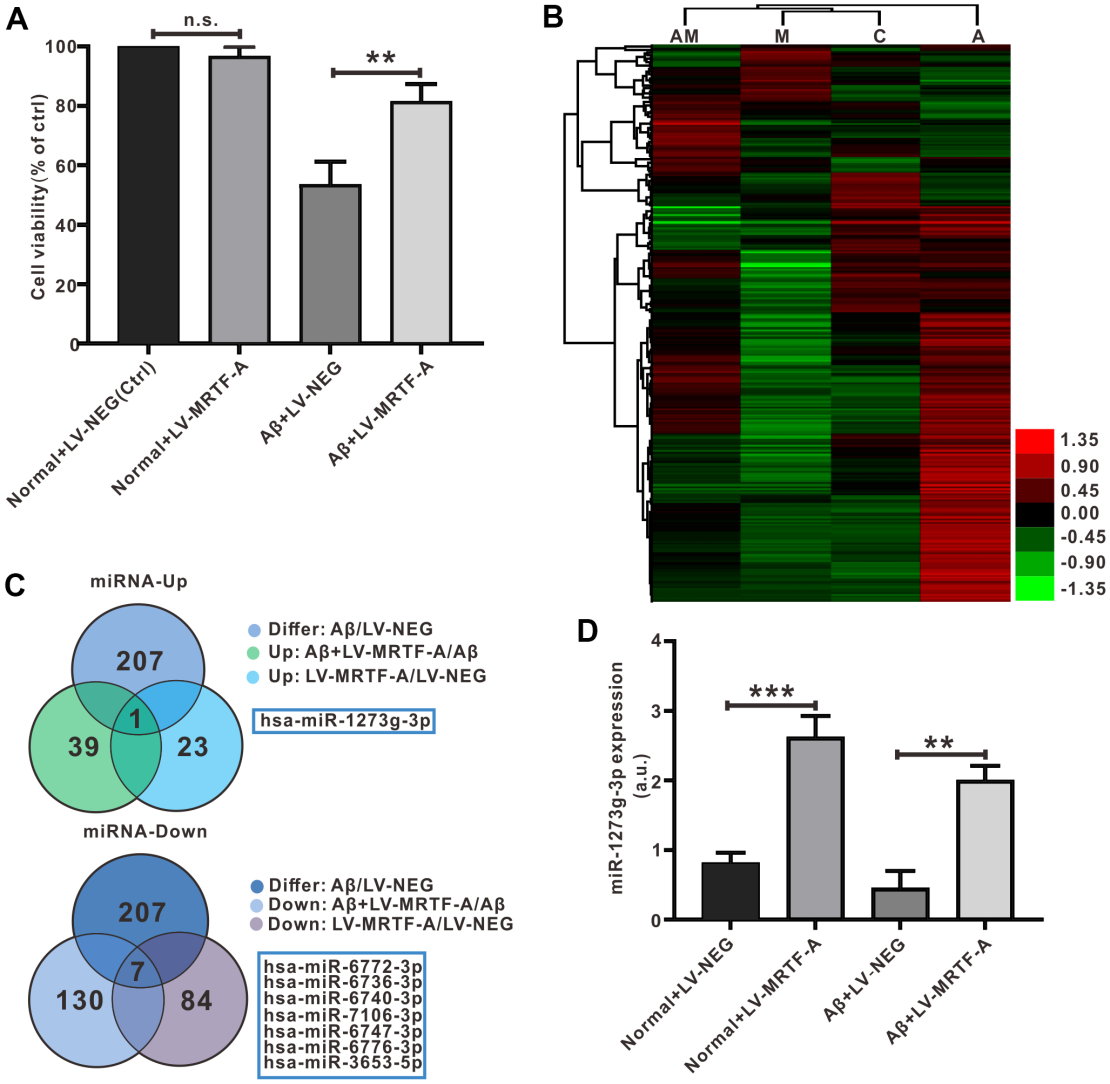
**Detection and verification of miRNA microarray in Aβ-treated SH-SY5Y cells.** (**A**) MRTF-A increased cell viability that had been reduced by Aβ_1-42_ exposure (n=6). (**B**) Microarray cluster analysis(n=3). AM: Aβ+LV-MRTF-A, M: normal+LV-MRTF-A, C: normal+LV-NEG, A: Aβ+LV-NEG. (**C**) Venn plots showed DEMs. (**D**) The miR-1273g-3p level was analyzed by RT-PCR. Data represent means ± SEM of 3 independent experiments. *P < 0.05, **P < 0.01 and ***P < 0.001 (one-way ANOVA, Tukey’s Multiple Comparison Test).

To test the mechanism of MRTF-A on autophagy, transfection of Aβ-treated SH-SY5Y cells with LV-MRTF-A or its negative carrier were subjected to miRNA microarray analysis, and normal was as control. As shown in Figure 5A, compared with Aβ_1-42_ treatment group, transfection of LV-MRTF-A increased cell viability (Figure 5A). The microarray data that there were 207 differential expressed miRNAs (DEMs). Next, the DEMs underwent Venn analysis (Figure 5B, 5C). A total of 8 significantly DEMs were determined, including one up-regulated and seven down-regulated. To confirm the regulatory effect of MRTF-A on miR-1273g-3p, Aβ-treated SH-SY5Y cells were transfected with LV-MRTF-A. As shown in Figure 5D, LV-MRTF-A significantly increased miR-1273g-3p level (≥ 4.2 times) in normal or Aβ-treated SH-SY5Y cells (Figure 5D).

### mTOR was the target gene of miR-1273g-3p

**Figure 6:**
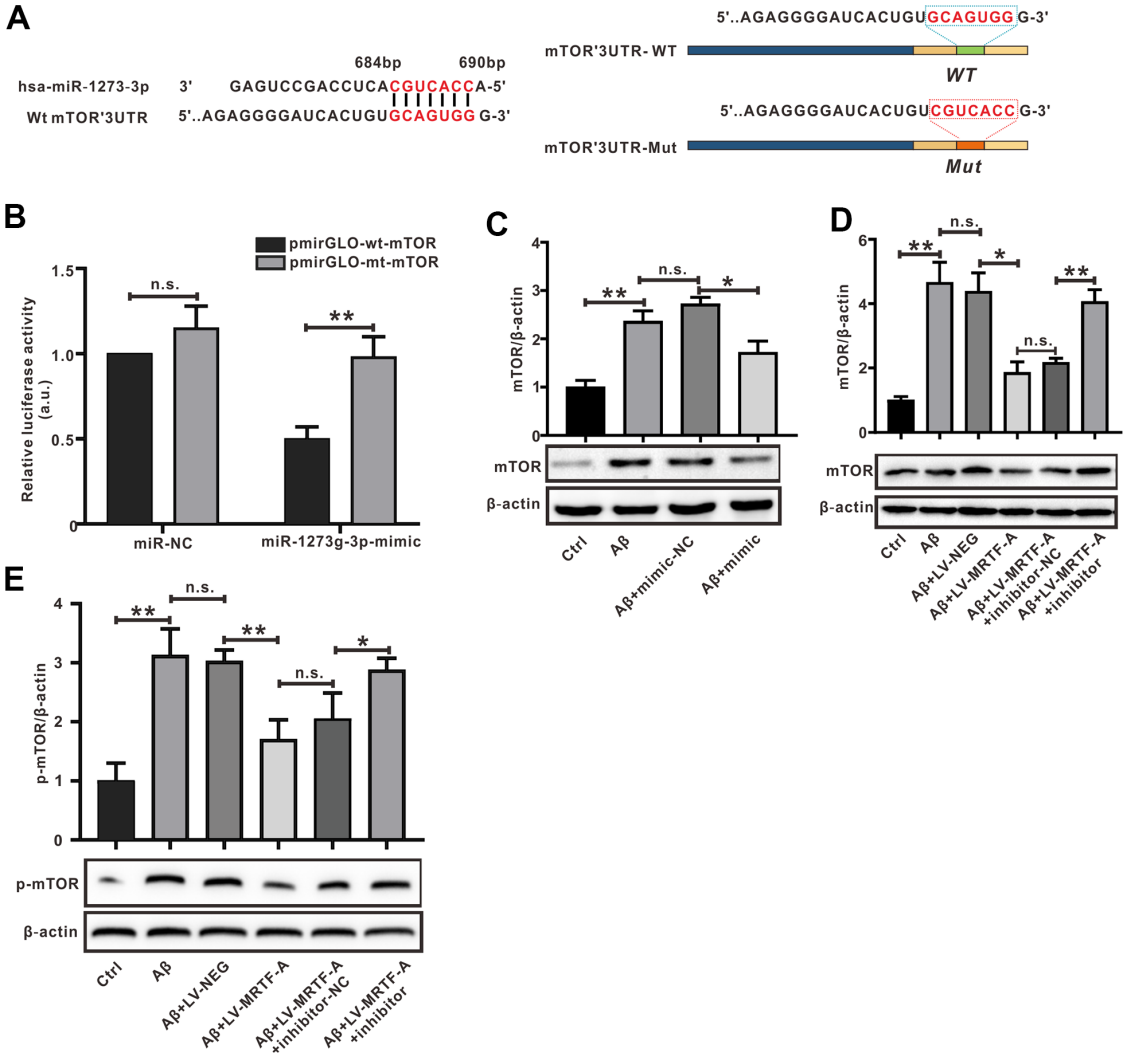
**mTOR was the target of miR-1273g-3p.** (**A**) A complementary pairing area or mutant area was shown between miR-1273g-3p and 3′-UTR mTOR with the wild type or mutant type, respectively. (**B**) The dual luciferase reporter assay. (**C**–**E**) The protein expression of mTOR and p-mTOR was detected by western blot. Data represent means ± SEM of 3 independent experiments. *P < 0.05, **P < 0.01 and ***P < 0.001 (one-way ANOVA, Tukey’s Multiple Comparison Test).

In order to explore the mechanism of miR-1273g-3p on Aβ-induced neuronal damage, miRBase and Targetscan were used to predict the target genes of miR-1273g-3p. We obtained the sequence of miR-1273b-3p and mTOR 3’-UTR through miRbase and found the complementary pairing region between miR-1273g-3p and mTOR sequence in the 3’-UTR of Homo sapiens (Hsa) (Figure 6A). The analysis of gene function Gene Ontology and Kyoto Encyclopedia of Genes and Genomes database suggested that the binding sites of miR-1273g-3p enriched autophagy genes, and mTOR was one of the most potential target genes (Supplementary Figure 3). Bibiserv2 software was also used to analyze 3’-UTR Hybrid energy analysis between miR-1273g-3p and mTOR. The results showed that the free energy was lower (-46.7 kcal/mol), speculating that both binding sites may have strong binding ability and produce its high efficiency (Supplementary Figure 4).

To verify whether mTOR was a target gene of miR-1273g-3p, the dual luciferase reporter assay was performed (Figure 6B). The luciferase activity of mTOR-wt luciferase reporter vector was notably suppressed in response to transduction of miR-1273g-3p mimic when compared with the mimic-NC group (*P*< 0.05), indicating a direct combination between miR-1273g-3p and mTOR 3′-UTR. Western blot analysis confirmed that transduction of miR-1273g-3p mimic in Aβ-treated SH-SY5Y cells negatively regulated mTOR protein expression by targeting mTOR 3’-UTR (Figure 6C); meanwhile, transfection of LV-MRTF-A decreased the expression of mTOR and its phosphorylated (p-mTOR) proteins; however, this effect was abrogated by co-transfection with miR-1273g-3p inhibitor (Figure 6D, 6E).

### miR-1273g-3p/mTOR axis involved in the role of MRTF-A on Aβ-induced autophagy impairment

**Figure 7:**
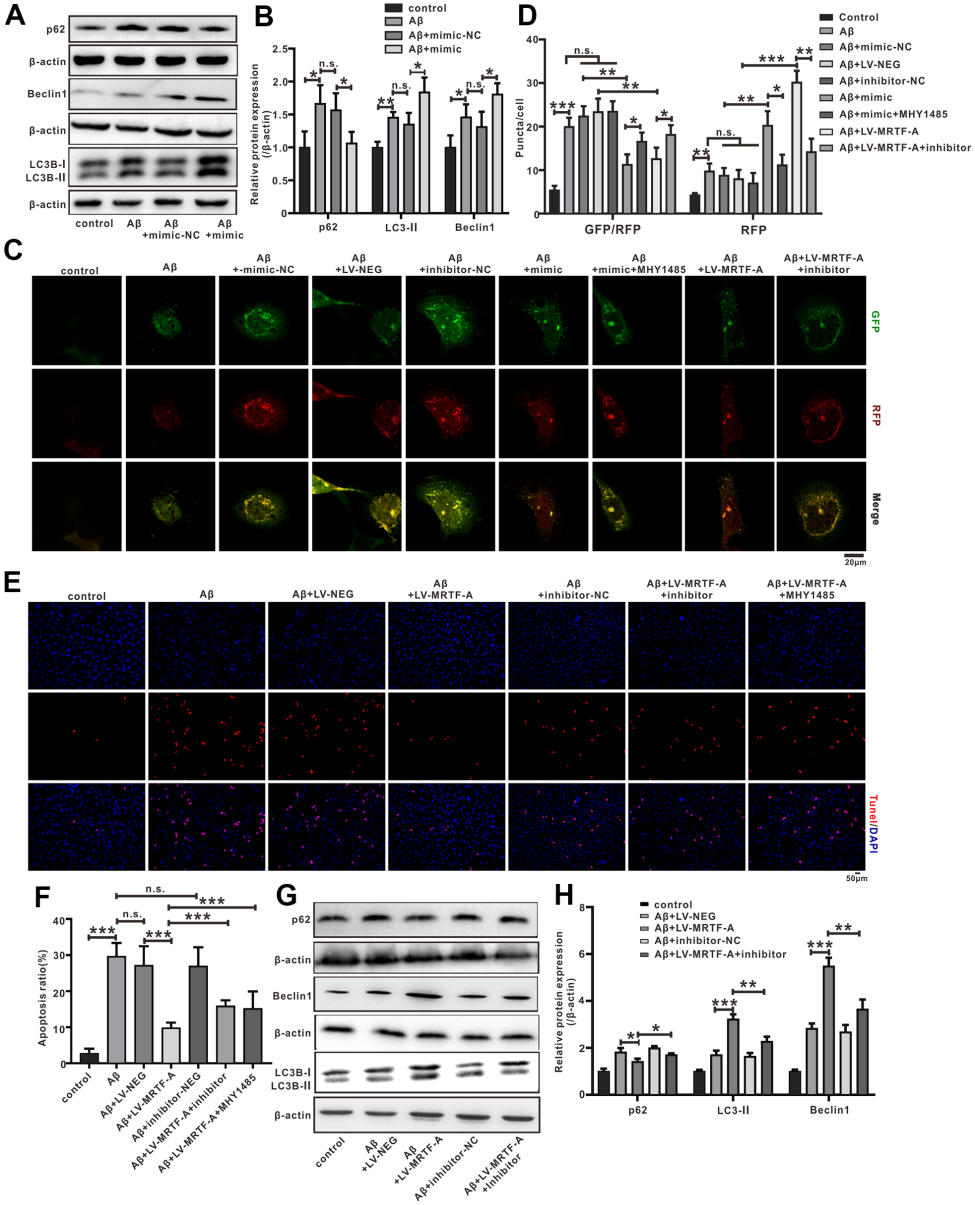
**The miR-1273g-3p/mTOR axis involved in MRTF-A against Aβ-induced autophagy impairment and neuronal apoptosis in SH-SY5Y cells.** (**A**, **B**) LC3II, Beclin1 and p62 level was upregulated by miR-1273g-3p. (**C**, **D**) The autophagy flux analysis using Ad-mCherry-GFP-LC3B-tagged protein. (**E**, **F**) TUNEL staining for cell apoptosis. (**G**, **H**) LC3II, Beclin1 and p62 level was upregulated by MRTF-A. Data represent means ± SEM of 3 independent experiments. *P< 0.05, **P < 0.01 and ***P < 0.001 (one-way ANOVA, Tukey’s Multiple Comparison Test).

To explore the effect of miR-1273g-3p on Aβ-induced neuronal autophagy impairment. Cells were transfected with miR-1273g-3p mimic or its negative vector for 48 hours and then exposed to Aβ_1-42_. 24 hours later, western blotting showed that miR-1273g-3p significantly increased the expression of LC3II and Beclin1 and decreased the level of p62 compared with the Aβ+mimic-NC group (Figure 7A, 7B). Autophagy flow analysis showed that normal SH-SY5Y cells exhibited basal autophagy; however, the cells subjected to Aβ_1-42_ exhibited increased autophagosomes and few autolysosomes. Upregulating of miR-1273g-3p significantly increased the formation of autolysosomes compared with that in Aβ+mimic-NC, but this effect was declined by MHY1485(an agonist of mTOR) (Figure 7C). These results indicated that miR-1273g-3p reversed Aβ-induced autophagy impairment partially dependent on mTOR pathway.

To further confirm whether miR-1273g-3p is involved in the activation of autophagy by MRTF-A, the cells were transduced with LV-MRTF-A and co-transfected with or without miR-1273g-3p inhibitor. As expected, compared with the Aβ or Aβ+LV-NEG group, transduction of LV-MRTF-A significantly increased the formation of autolysosomes in Aβ-treated cells, and this effect was partially eliminated by co-treatment with miR-1273g-3p inhibitor (Figure 7C). Western blotting showed that overexpression of MRTF-A increased the expression of LC3II and Beclin1 and decreased p62 levels, which was also partially reversed by miR-1273g-3p inhibitor (Figure 7D, 7E). Accordingly, overexpression of MRTF-A showed a significant inhibitory effect on apoptosis, but in SH-SY5Y cells treated with Aβ, this effect was partially reversed by co-treatment with miR-1273g-3p inhibitor (Figure 7F, 7G).

## DISCUSSION

In the present study, we investigated the effects and the underlying mechanism of MRTF-A on Aβ-induced neurotoxicity and autophagy dysfunction *in vivo* and *in vitro* Alzheimer models. Our current research shows that there is a decreasing of MRTF-A protein in the hippocampus of Tg2576 mice, which is related to its learning and memory impairment. Overexpression of MRTF-A improved memory impairment and cognitive function in Tg2576 AD mouse model.

Alzheimer’s disease is characterized by accumulation of amyloid beta (Aβ) and tau protein in the brain, leading to neurodegeneration with progressive cognitive decline [41, 42]. Tg2576 mice, as AD experimental models, exhibit Aβ accumulation and cognitive deficits, and overexpress human amyloid precursor protein (hAPP) subtype 695 containing the Swedish double mutation [31, 32]. In Aβ-treated cells and animal models, the level of APP and BACE1 is involved in Aβ production, leading to increased Aβ -induced neuronal toxicity. We firstly demonstrated that upregulating MRTF-A significantly decreased the Aβ plaques accumulation and reduced the protein expression of APP and BACE1 and alleviated hippocampal neuronal damage in Tg2576 mice.

Interestingly, the analysis of co-expression network construction revealed that MRTF-A function was related to autophagy related genes such as Beclin1 and ATG5 and so on, suggesting MRTF-A involving in autophagy process. Accumulating evidence showed that the impairment in the autophagy-lysosome system disturbs the turnover of other molecules associated with Alzheimer’s disease, which may also contribute to the neuronal dysfunction in Alzheimer’s disease [43, 44]. Several lines of evidence have suggested that a tight relationship between Aβ production and mTOR in AD. Activation of PI3K/Akt/mTOR increased amyloid precursor protein synthesis and deposition, in part by inhibiting autophagy mediated Aβ clearance, while restoration of autophagy reverses cognitive decline and ameliorated Aβ pathology [37, 45]. Beclin1 and LC3 initiate autophagogenesis by interacting with ATG3/ATG7 effectors. The P62 protein is an adaptor that binds LC3 on autophagosomes to ubiquitinated proteins on cargo to recruit autophagosomes, which is continuously degraded by autophagy-lysosomal system. Autophagy dysfunction leaded to p62 accumulation [46]. In the present study, autophagy dysfunction was also found in the hippocampal region of Tg2576 mice, which was partially restored by upregulating MRTF-A, the similar results were confirmed in SH-SY5Y cells after exposed to Aβ_1-42_. We found that overexpression of MRTF-A could decreased p62 protein expression and increased Beclin1 protein level both *in vivo* and *in vitro*. *In vitro* autophagy flux analysis also showed that MRTF-A can rescue Aβ-induced autophagy damage, which is the reason for the inhibitory effect of MRTF-A on Aβ-induced apoptosis of SH-SY5Y cells.

MiRNAs is reported that miRNAs are involved in a variety of human diseases [47], including in the autophagy process following AD. Increasing evidence has shown that miRNAs are strongly associated with AD [48]. Because there is some correlation between miRNA and autophagy [49]. We postulated that the effect of MRTF-A on autophagy in the pathological process of AD was associated with miRNAs. For the purpose, we next performed miRNAs expression profile to predict the differential expressed miRNAs (DEMs) which involved in MRTF-A against Aβ-induced neuronal damage. The miR-1273g-3p was identified as one of the most significant DEMs. Bioinformatics analysis shows that miR-1273g-3p is enriched in autophagy genes, and mTOR is one of the most potential target genes. The mTOR gene, a negative regulator of autophagy, can play the role of “gatekeeper” for autophagy initiation, which plays a central role in autophagy regulation and also the central link of PI3K/AKT signaling pathway [50–53]. Furthermore, the binding free energy between miR-1273g-3p and mTOR was low, speculating the strong binding ability. The results of dual luciferase reporter assay and western blot showed that miR-1273g-3p negative regulated the level of mTOR and p-mTOR protein. The relationship between MRTF-A and miR-1273g-3p/mTOR axis prompted us to speculate that miR-1273g-3p is involved in the effect of MRTF-A on autophagy. As predicted, the use of miR-1273g-3p alone increased the protein expression of LC3II and Beclin1 and decreased p62 expression of, and at the same time enhanced the autophagy flux, and this effect could be eliminated by MHY1485 (mTOR agonist), indicating that miR-1273g-3p can rescue Aβ-induced autophagy damage partially depends on the mTOR pathway. In addition, we also found that MRTF-A-mediated recovery of autophagy impairment by promoting miR-1273g-3p/mTOR axis helps protect MRTF-A from neuronal damage.

## CONCLUSIONS

**Figure 8:**
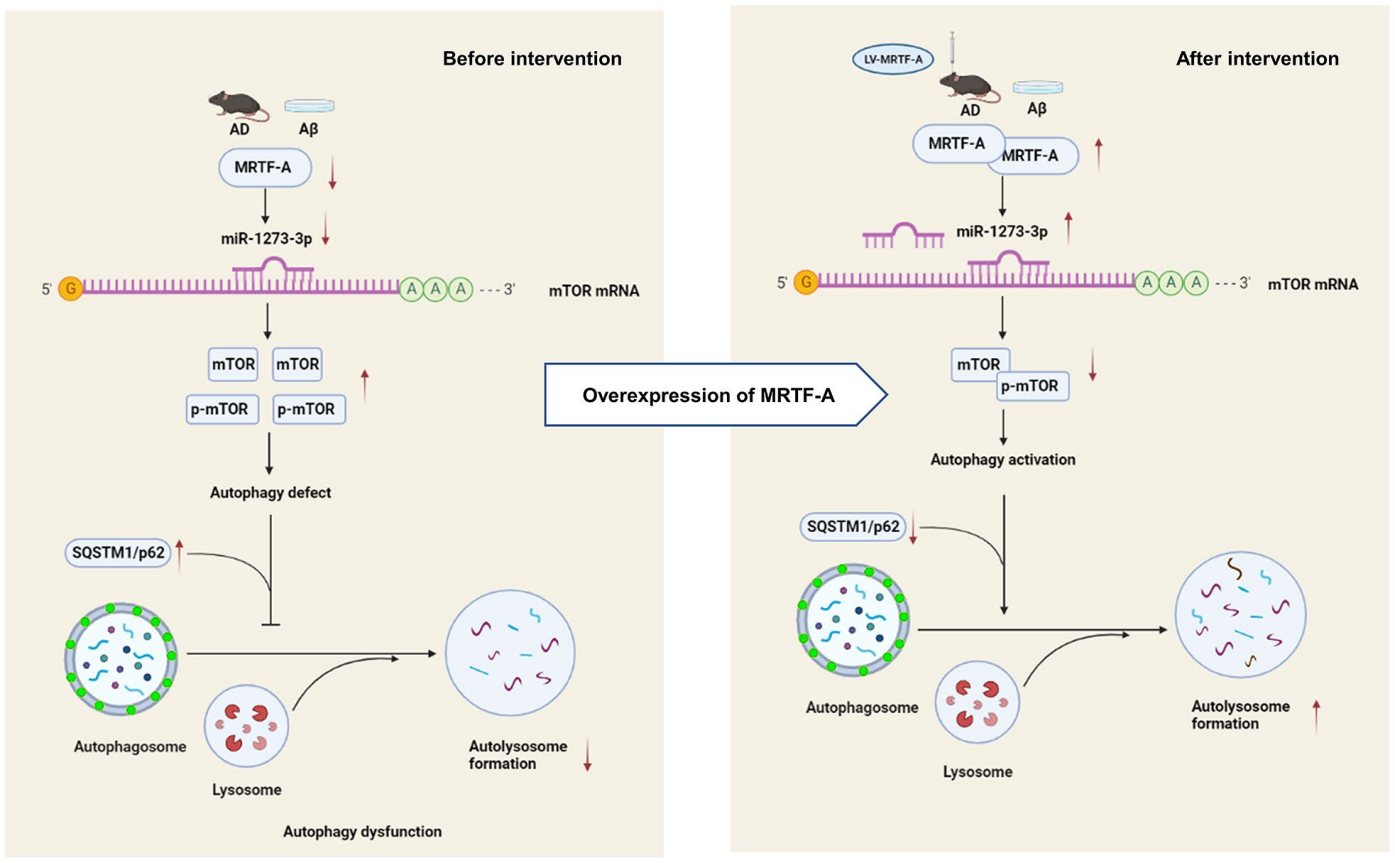
The schematic description of the mechanism of MRTF-A/miR-1273g-3p/mTOR axis in regulating autophagy in Alzheimer models.

Our study shows that MRTF-A improves cognitive deficit of Alzheimer mice and alleviated Aβ-induced neurotoxicity *in vivo* and *in vitro*. Further, MRTF-A rescues the Aβ-induced autophagy impairment via regulating miR-1273g-3p/mTOR axis, which maybe contributes to the protective effect of MRTF-A against Aβ-induced neurotoxicity. Therefore, upregulation of MRTF-A or activation of miR-1273g-3p/mTOR axis may serve as a potential therapeutic target in AD treatment. (Summarized in Figure 8).

## Supplementary Material

Supplementary Figures
