# Supplementary material for: MRTF-A-mediated protection against amyloid-β-induced neuronal injury correlates with restoring autophagy via miR-1273g-3p/mTOR axis in Alzheimer models
Source: Aging (Albany NY). 2022 May 23;14(10):4305–25. doi: 10.18632/aging.203883 (PMC9186769; doi:10.18632/aging.203883)
Supplement: Supplementary Figures [file aging-14-203883-s001.pdf]

SUPPLEMENTARY FIGURES

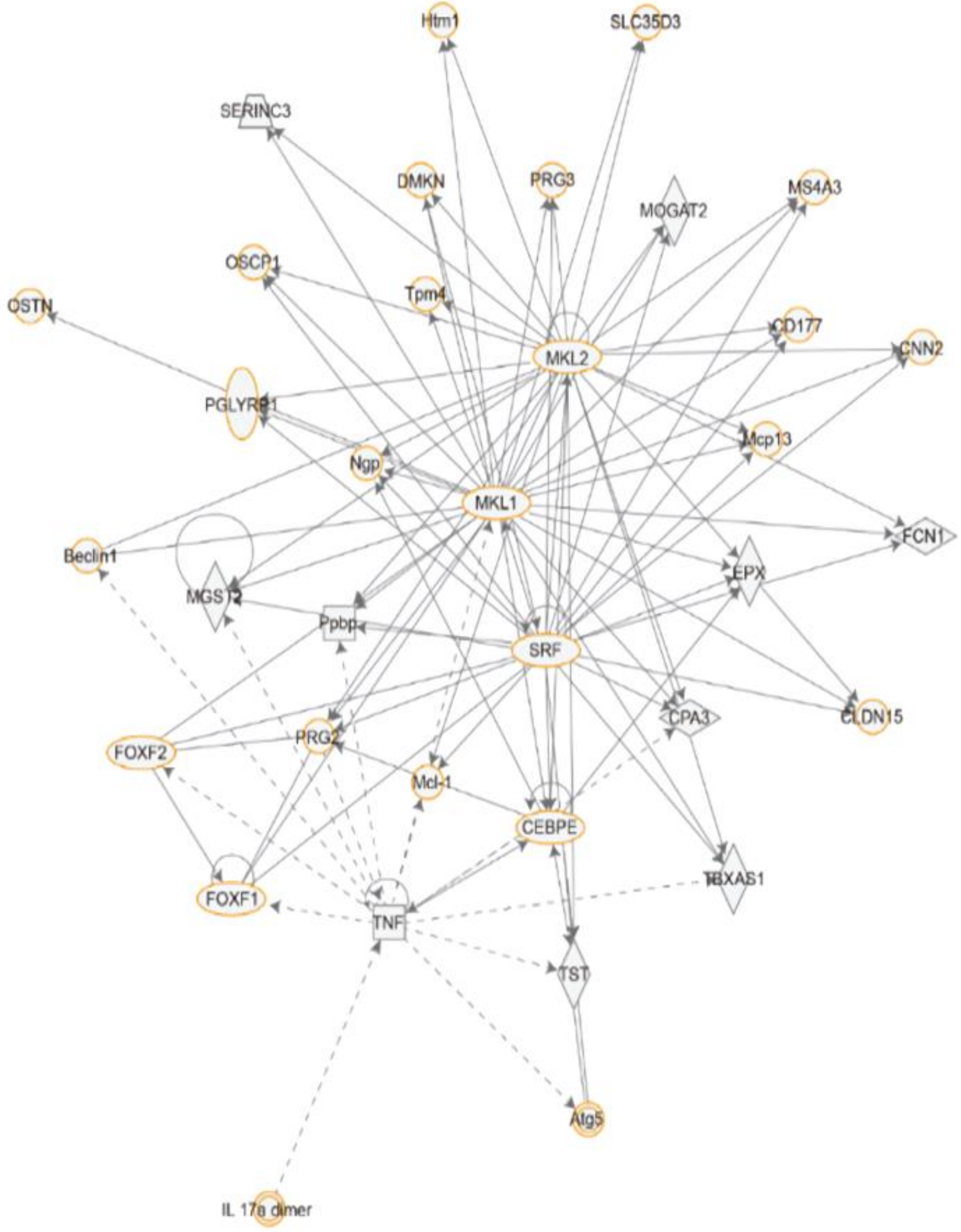

Supplementary Figure 1. The functional and high connected hubs varied about MRTF-A analyzed by IPA tool.

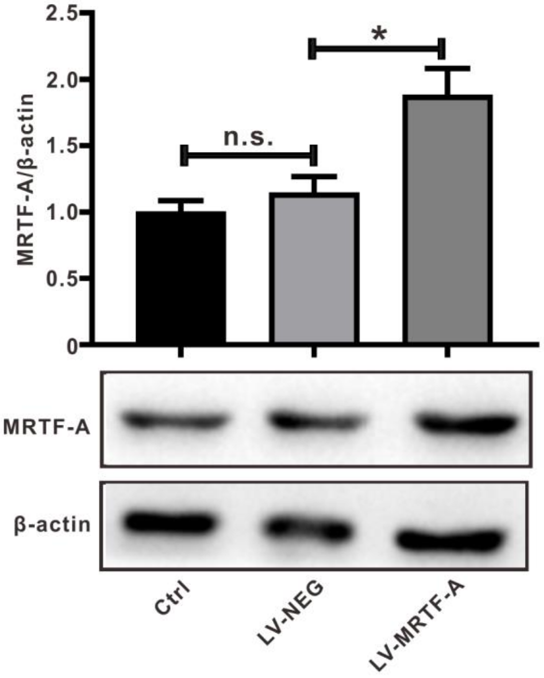

Supplementary Figure 2. MRTF-A protein expression was determined by western blot after transduction with LV-MRTF-A or LV-NEG for 48 hours in SH-SY5Y cells. **P*< 0.05, ***P* < 0.01 and ****P* < 0.001 (one-way ANOVA, Tukey's Multiple Comparison Test).

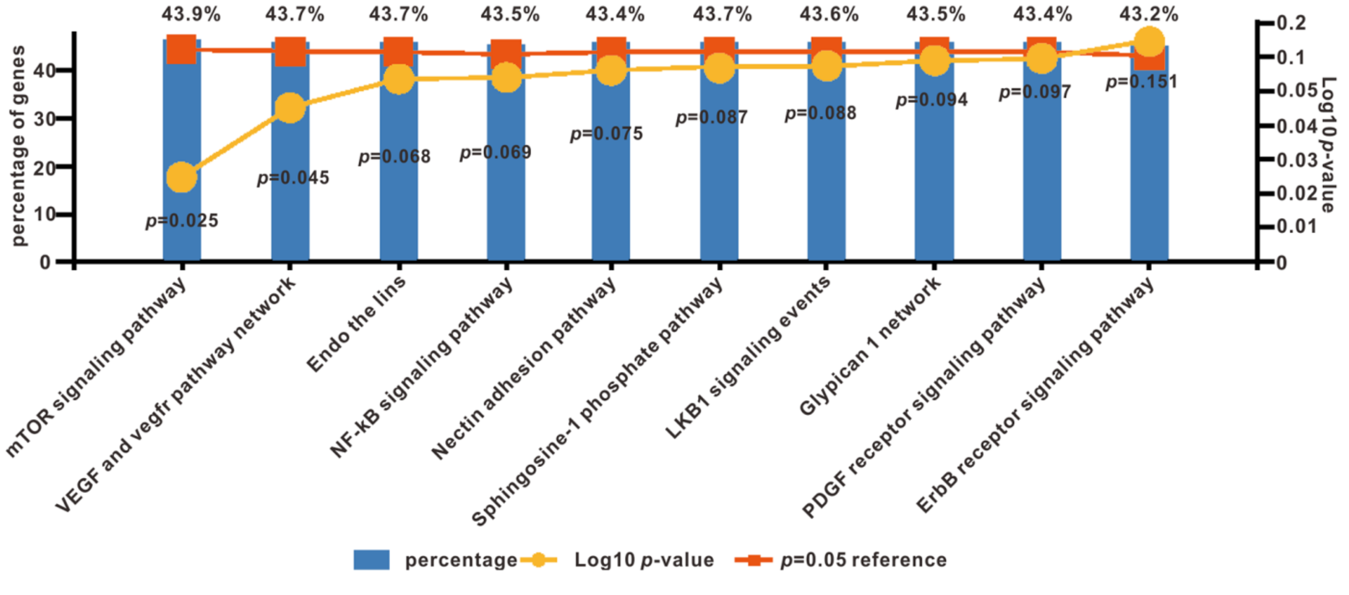

Supplementary Figure 3. The gene function Gene Ontology (GO) analysis and Kyoto Encyclopedia of Genes and Genomes (KEGG) database analysis to identify the involved enriched pathways.

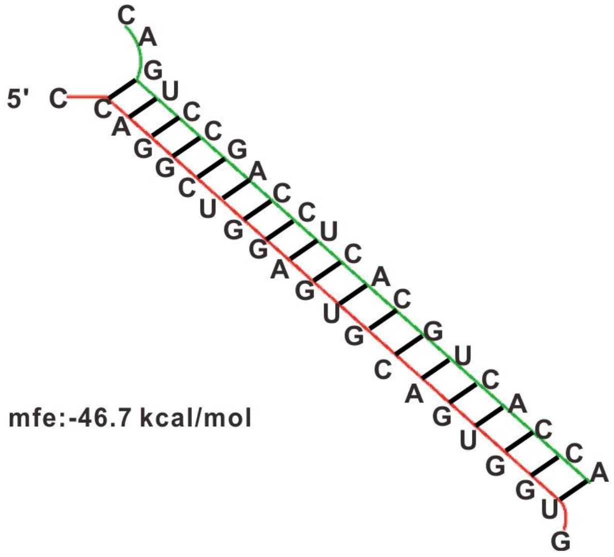

Supplementary Figure 4. Bibiserv2 software was used to analyze 3’-UTR Hybrid energy analysis between miR-1273g-3p and mTOR.
